# Quadriceps muscle status after medial-pivot versus ultracongruent total knee arthroplasty using localized bioimpedance analysis: a randomized exploratory study

**DOI:** 10.1186/s42836-026-00429-z

**Published:** 2026-09-07

**Authors:** Glòria Pedemonte-Parramón, Lexa Nescolarde, Ester García-Oltra, Vicente López, Lika Dzidzishvili, José A. Hernández-Hermoso

**Affiliations:** 1 Department of Orthopedic Surgery and Traumatology, Hospital Universitari Germans Trias i Pujol, Barcelona, Badalona 08916 Spain; 2 Department of Surgery, Universitat Autònoma de Barcelona, Edifici U, Campus UAB, 08913-Bellaterra, 08916 Barcelona, Spain; 3 Department of Electronic Engineering, Universitat Politècnica de Catalunya-BarcelonaTech, Barcelona East School of Engineering (EEBE), 08034 Barcelona, Spain

**Keywords:** Total knee arthroplasty, Medial pivot, Ultracongruent, Localized bioelectrical impedance, Quadriceps muscle recovery, Randomized trial

## Abstract

**Background:**

Implant design in total knee arthroplasty (TKA) may influence postoperative biomechanics and muscle recovery. Ultracongruent (UC) and medial pivot (MP) designs aim to enhance anteroposterior stability and reproduce physiological knee kinematics, yet comparative data remain scarce, and muscle recovery has rarely been directly evaluated. Localized bioelectrical impedance analysis (L-BIA) provides an objective method to assess skeletal muscle status, but its application to compare muscle recovery between different TKA implant designs has not been previously investigated.

**Methods:**

A prospective, randomized, patient-blinded study was conducted between 2022 and 2024, including 40 patients undergoing primary TKA for advanced knee osteoarthritis. Patients were randomly assigned to receive either a medial-pivot (MP) or an ultracongruent (UC) prosthesis. The final analysis included 30 patients (15 per group). The primary outcome was postoperative quadriceps muscle recovery between implant groups, assessed longitudinally by reactance (Xc) of the rectus femoris, vastus medialis, and vastus lateralis using localized bioelectrical impedance analysis (L-BIA). Assessments were performed preoperatively and at 6 and 12 months postoperatively. Secondary outcomes included resistance (R), WOMAC, Knee Society Score, range of motion, and radiographic parameters. Temporal changes and between-group differences were analysed using repeated-measures and covariance-based statistical models.

**Results:**

Demographic and preoperative characteristics were comparable between groups. Resistance values remained stable across all time points with no differences between implant designs. Reactance showed a transient decrease at 6 months followed by recovery at 12 months in both groups, with no significant differences between groups. ANCOVA demonstrated that baseline reactance was associated with 12-month reactance values (all *p* < 0.001), whereas implant design had no significant effect. The clinical relevance of this finding remains uncertain. Radiographic parameters, postoperative range of motion, and clinical outcomes improved significantly after surgery in both groups without significant differences between MP and UC designs.

**Conclusion:**

No significant differences in quadriceps recovery were observed between MP and UC implants during the first postoperative year. Baseline muscle status was associated with postoperative L-BIA outcomes, whereas no significant differences were observed between implant designs.

**Supplementary Information:**

The online version contains supplementary material available at https://doi.org/10.1186/s42836-026-00429-z.

## Introduction

Total knee arthroplasty (TKA) is the treatment of choice for patients with advanced gonarthrosis who do not respond to conservative management [1]. Several prosthetic designs exist, each based on different biomechanical principles and stability strategies [2, 3].

Traditionally, the most widely used designs were cruciate-retaining (CR) implants, which preserve the posterior cruciate ligament (PCL), and posterior-stabilized (PS) implants, which replace its function through a post-cam mechanism. However, both designs have inherent drawbacks. CR implants require a functionally competent and appropriately tensioned PCL, making ligament balancing technically demanding and potentially resulting in altered knee kinematics if the PCL is too tight or too lax. In contrast, PS implants increase prosthetic constraint and polyethylene wear due to post-cam interaction, with potential complications including post-cam dislocation, patellar clunk syndrome, and greater bone resection [4–6].

To address some of the limitations of traditional designs, newer insert designs have been developed with different biomechanical objectives. [4, 7]. Ultracongruent (UC) inserts enhance anteroposterior stability through greater articular conformity without a post-cam mechanism [4, 6], whereas medial-pivot (MP) inserts aim to maintain a stable medial compartment with controlled lateral rollback during flexion [7–9].

The differences in implant biomechanics may result in distinct kinematic patterns and muscle activation demands, which can be one of the factors that may influence postoperative quadriceps recovery.

Given that patients with knee osteoarthritis already exhibit preoperative quadriceps atrophy due to pain, arthrogenic muscle inhibition, and reduced functional loading [10–14], these alterations may also translate into structural muscle changes, including increased intramuscular fatty infiltration [14, 15]. Moreover, considering that quadriceps atrophy often worsens after surgery [10–12], it is essential that prosthetic design does not further exacerbate this condition but ideally facilitates more efficient muscle recovery.

Despite the widespread use of both designs, UC and MP, comparative data regarding their influence on muscle recovery remain scarce, and most studies have focused on clinical scores or kinematic analyses rather than direct muscle assessment [4, 9, 16–19].

Localized bioelectrical impedance analysis (L-BIA) is a direct, non-invasive, real-time technique for objectively assessing skeletal muscle status through measuring tissue electrical properties. In particular, resistance (R) is related to tissue hydration, and reactance (Xc) reflects muscle cell integrity [20, 21].

Previous studies have demonstrated the ability of L-BIA to detect structural muscle changes in diverse clinical settings, including wound healing, neuromuscular disorders, and sports-related muscle injuries [22–28].

Recently, Pedemonte-Parramón et al. demonstrated that serial L-BIA measurements are sensitive to longitudinal changes in quadriceps muscle recovery after TKA, with reactance (Xc) decreasing during the early postoperative period and returning to baseline or higher values by 12 months, whereas resistance (R) remained relatively stable [29]. These findings support the use of L-BIA as an objective tool for monitoring postoperative quadriceps muscle recovery.

Considering that both MP and UC designs aim to improve knee stability through different biomechanical principles, this study aimed to evaluate whether postoperative quadriceps muscle recovery, assessed using localized bioimpedance, differed between the two implant types. However, the potential influence of implant design on muscle recovery remains unknown, and no studies have directly compared MP and UC TKA using objective tissue-level muscle assessment. Given the central role of quadriceps recovery in restoring gait, functional independence, and return to activities of daily living after TKA, understanding whether muscle recovery differs according to implant design could provide clinically relevant information [10, 12, 15, 30, 31]. Such information could contribute to more individualized implant selection, optimize postoperative rehabilitation strategies, and improve preoperative patient counseling regarding expected functional recovery.

Moreover, conventional clinical outcome scores primarily reflect pain, function, and patient-reported outcomes rather than the biological status of the quadriceps muscle. Consequently, they may not detect differences in postoperative muscle recovery, highlighting the need for objective methods capable of directly assessing muscle tissue.

This study aimed to compare quadriceps bioelectrical muscle recovery, assessed by L-BIA, in patients undergoing TKA with two implant designs: UC and MP prostheses. Secondary objectives were to compare postoperative clinical outcomes and radiographic measurements between both groups to evaluate whether differences in muscle recovery could be explained by differences in implant positioning.

## Methods

### Study design

A prospective, randomized, patient-blinded, investigator-initiated study was conducted at a tertiary referral hospital between 2022 and 2024. The study was approved by the Institutional Review Board in accordance with the principles of the Declaration of Helsinki (Approval No. PI-20-045). Written informed consent was obtained from all participants before surgery. This was an investigator-initiated, non-commercial academic study conducted within an institutional research setting. The trial was not prospectively registered.

The primary study outcome was the comparison of postoperative quadriceps bioelectrical recovery between the ultracongruent (UC) and medial-pivot (MP) groups, assessed longitudinally by serial reactance (Xc) measurements of the rectus femoris, vastus medialis, and vastus lateralis using localized bioelectrical impedance analysis (L-BIA). Resistance (R), clinical outcomes, and radiographic measurements were considered secondary outcomes.

### Sample size

Because no previous studies had compared prosthetic designs according to localized reactance (Xc), the sample size calculation was based on the best available evidence. Specifically, previously reported longitudinal physiological changes in muscle Xc were used as a surrogate estimate of the expected effect size, as no between-group estimates were available. Based on these data [29], reactance decreased by approximately 16% between the preoperative assessment and the 6-month follow-up (≈1.3 Ω). Assuming a standard deviation of 2.25 Ω, a two-sided alpha of 0.05, and 80% power, the estimated required sample size was 25 patients. Therefore, the calculation was based on a surrogate longitudinal effect rather than an expected between-group difference.

### Patient’s sample

**Fig. 1:**
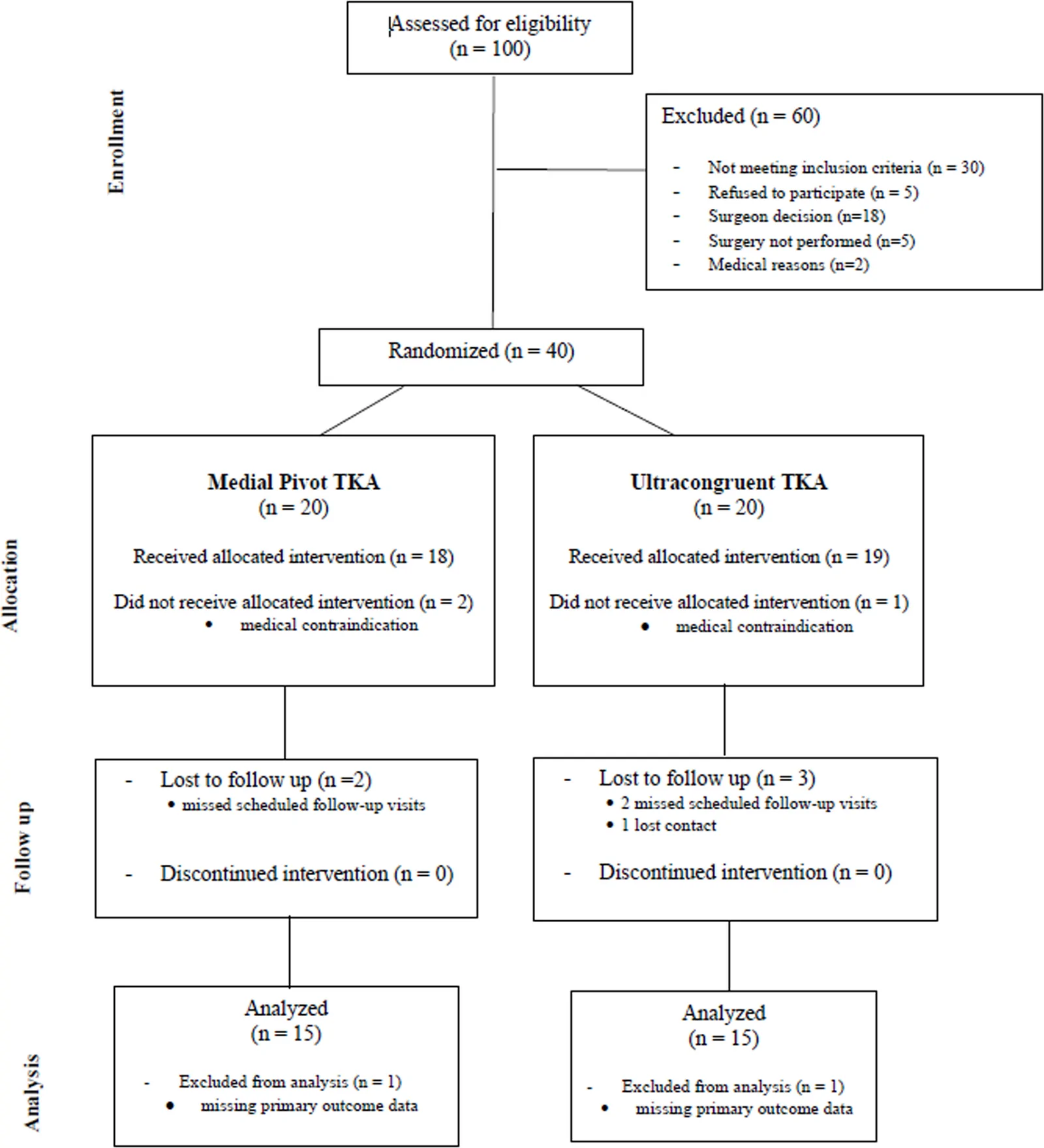
Patient’s flow diagram

A total of 40 patients scheduled to undergo primary total knee arthroplasty were enrolled and randomized in this study. The final analysis included 30 patients (15 in each group), as shown in the participant flow diagram (Fig. 1).

Patients were randomized in a 1:1 ratio using a computer-generated randomization list. The randomization sequence was generated and managed by the principal investigator, who was the only member of the study team with access to the allocation list. After providing written informed consent and agreeing to participate, each patient was assigned to the next available allocation on the randomization list using a simple numerical coding system linked to the two implant types (code 1 = Evolution®, MicroPort; code 2 = Columbus®, B. Braun Aesculap).

The allocation sequence was concealed from the operating surgeons and other study personnel until assignment; the operating surgeon was informed of the allocated implant the day before surgery. The study was conducted as a single-blind trial, with patients remaining blinded to implant allocation throughout the study. The principal investigator, who managed the randomization sequence and performed the ROM and L-BIA measurements, was not blinded to group allocation. Radiographic measurements were also performed by the principal investigator and were therefore not blinded. Moreover, implant design could potentially be inferred from the appearance of the postoperative radiographs. Physical therapists remained blinded to implant type, and statistical analyses were performed independently by an analyst blinded to group allocation.

Inclusion criteria comprised patients aged between 40 and 85 years with a diagnosis of primary knee osteoarthritis, classified as grade III or higher according to the Ahlbäck scale, presenting with severe pain and functional limitation unresponsive to conservative treatment for at least six months.

Exclusion criteria were: (1) patients deemed unsuitable for elective TKA because of severe cardiovascular or pulmonary disease or unacceptable anesthetic risk; (2) severe preoperative deformities exceeding 15° of varus or valgus; (3) polyarticular involvement of the lower limbs or lumbar spine leading to functional impairment; (4) previous ipsilateral knee surgery or any additional surgery during follow-up; (5) contralateral knee surgery; (6) diagnosis of neuromuscular or neurodegenerative disorders; (7) known metal hypersensitivity requiring a specific implant model; and (8) refusal to participate or withdrawal of informed consent.

### Patient data

Patients were evaluated preoperatively and at 6 and 12 months post TKA. Epidemiological data were recorded, including age, sex, operated side, history of previous knee or contralateral total knee replacement, body mass index (BMI), and American Society of Anesthesiologists (ASA) classification.

Whole-body bioimpedance variables (R/H and Xc/H) were collected at baseline to characterize the overall bioelectrical status of the study population and are presented only as descriptive baseline variables.

Knee range of motion (ROM) was assessed by an independent investigator using a standard goniometer (model 27,340, Gima, Gessate, Italy). The instrument’s axis was positioned over the knee joint center, with the stationary arm aligned along the femur toward the greater trochanter and the movable arm aligned with the fibular shaft toward the lateral malleolus.

### Clinical assessment

Clinical outcomes were evaluated preoperatively and at 6 and 12 months post-TKA using two validated scores: Western Ontario and McMaster Universities Osteoarthritis Index (WOMAC) [32] and the Knee Society Score (KSS) [33].

### Radiological analysis

Standing full-weight-bearing lower limb radiographs, as well as true lateral (posterior condyle superimposition at 30° flexion) and axial knee views (30°), were obtained preoperatively and at 6 and 12 months postoperatively following a standardized protocol to ensure reproducibility and minimize rotational bias [34].

Joint line (JL) was measured on anteroposterior radiographs, whereas posterior condylar offset (PCO), tibial slope (TS), and patellar height (PH) were assessed on lateral radiographs. Measurements were obtained using a semiautomatic calibrated system (ALMA®). Calibration was performed using a radiopaque marker of known diameter positioned on the medial aspect of the distal tibia and aligned with the coronal plane of the limb, following the general principle of marker-based radiographic calibration for magnification correction [35, 36]. Because of its standardized position close to the tibia, the marker provides a reproducible reference for magnification correction while minimizing the influence of rotational differences between serial radiographs. All radiographs were analyzed by an independent observer. Because the implant design could be identified on postoperative radiographs, blinding to implant allocation was not feasible. To assess intra-observer reliability, each measurement was repeated after six weeks (ICC = 0.97).

JL height was measured using the Fibular Head method [37–39] and the Adductor Tubercle technique [40]. Patellar height was calculated using the Insall–Salvati [41] and Caton–Deschamps indices [42]. PCO was defined as the distance from the posterior femoral cortex to the posterior condylar margin [43], and the PCO ratio (PCOR) was calculated by dividing PCO by the maximum anteroposterior diameter of the distal femur [44]. Tibial slope (TS) was measured by first defining the anatomical tibial axis and then drawing a line along the medial tibial plateau. Tibial slope was measured as the angle between the medial tibial plateau and a line perpendicular to the anatomical tibial axis [45].

Positional changes were calculated as the difference between postoperative and preoperative values, with positive values indicating an increase. For TS, posterior inclination was recorded as positive.

### Surgical procedure

All procedures were performed by experienced orthopaedic surgeons following a standardized surgical protocol. Surgeons were considered experienced if they were senior consultants with at least 5 years of independent knee arthroplasty practice and an annual volume of ≥ 50 primary total knee arthroplasties. Four surgeons met these criteria and participated in the study. All surgeons routinely performed both UC and MP TKA, and implant allocation was determined by the study randomization schedule, independent of the operating surgeon. The distribution of procedures across the four surgeons was comparable between the MP and UC groups (8/7, 2/2, 4/5, and 1/1 MP/UC procedures, respectively).

Antibiotic prophylaxis consisted of 2 g of cefazolin administered 30–60 min before incision, or 900 mg of clindamycin in patients with penicillin allergy. A pneumatic tourniquet was routinely used.

TKA was performed using a mechanical alignment strategy and a measured resection technique incorporating bone-cutting and gap-balancing principles. Two implant designs (Evolution™ and Columbus™) were randomly assigned. Surgical exposure was achieved through an anterior midline incision with medial parapatellar arthrotomy and routine posterior cruciate ligament resection.

The tibial cut was performed using an extramedullary guide perpendicular to the mechanical axis in both groups. The final posterior tibial slope was determined by the implant-specific polyethylene insert geometry according to each manufacturer’s design. Femoral preparation used an intramedullary guide to obtain a distal femoral cut perpendicular to the mechanical axis, with femoral rotation set at 3° of external rotation relative to the posterior condylar axis. After completion of the bone resections, flexion and extension gaps were assessed using spacer blocks to verify balanced ligament tension. Selective soft-tissue releases were performed when necessary to obtain symmetrical and balanced gaps according to a standardized surgical technique.

Patellar resurfacing was performed in all cases, and all components were cemented using antibiotic-loaded bone cement. The tourniquet was released before closure, no drains were used, and postoperative thromboprophylaxis consisted of low-molecular-weight heparin (40 mg daily) for 30 days.

### Postoperative rehabilitation protocol

All participants followed the same standardized rehabilitation program developed by the Department of Rehabilitation, aimed at restoring knee function after TKA. Passive range-of-motion exercises began on the day of surgery, and patients were encouraged to sit and ambulate as tolerated from the first postoperative day. Early joint mobilization was supported using a continuous passive motion device (Kinetech™).

From the second postoperative day, patients attended daily supervised physiotherapy sessions lasting approximately 30–45 min. The rehabilitation program included range-of-motion exercises, progressive muscle strengthening, supervised gait training in parallel bars followed by ambulation with crutches, balance training, and stair negotiation (ascending and descending). Although all patients followed the same standardized institutional rehabilitation protocol, exercise progression was individualized according to each patient’s pain tolerance, range of motion, and functional recovery.

Hospital discharge typically occurred on postoperative day three or four, once patients had achieved independent ambulation with walking aids, safe stair negotiation (ascending and descending), and adequate pain control.

Following discharge, patients continued a structured home-based rehabilitation program under physiotherapist supervision three times per week. Rehabilitation protocols were identical for both study groups, with no implant-specific modifications. Adherence to the rehabilitation program was not formally monitored.

### Localized bioimpedance

#### L-BIA measurements

Bioimpedance assessments were performed with patients in the supine position after a 15-min rest period. All measurements were obtained in the morning under standardized conditions at room temperature. Patients were instructed to avoid applying moisturizing creams to the lower limbs and to refrain from physical exercise before the assessment. No fasting was required. Electrodes were applied according to the standardized tetrapolar L-BIA protocol described by Nescolarde et al. [25, 26], which uses a pair of electrodes to deliver current and another pair to record voltage.

Electrode placement for each muscle followed a standardized anatomical landmark–based protocol [29]. For the rectus femoris (RF), electrodes were placed 5 cm distal to the anterior inferior iliac spine and 2 cm proximal to the superior pole of the patella. For the vastus lateralis (VL), electrodes were positioned 2 cm distal to the anterior aspect of the greater trochanter and 2 cm lateral and proximal to the superior pole of the patella. For the vastus medialis (VM), electrodes were placed 2 cm medial to the RF reference point and 1 cm medial and 2 cm proximal to the superior pole of the patella.

At each anatomical landmark, the current-injecting and voltage-sensing electrodes were placed adjacent to each other without overlap. The distance between the proximal and distal electrode pairs was determined by the anatomical dimensions of each muscle rather than by a fixed inter-electrode distance. These distances were recorded during the baseline assessment and reproduced at all subsequent follow-up visits to ensure identical electrode placement and measurement consistency for each participant. Localized bioimpedance analyses were performed using the raw resistance (R) and reactance (Xc) values, whereas whole-body bioimpedance measurements were additionally normalized to height (R/H and Xc/H) according to standard bioimpedance methodology.

L-BIA measurements were performed on both the operated and contralateral limbs. The contralateral limb was used as an internal reference to facilitate interpretation of postoperative muscle recovery over time.

#### L-BIA device

Tetrapolar bioimpedance measurements were obtained using a phase-sensitive impedance device (BIA 101 Anniversary, Akern-Srl, Florence, Italy), delivering a constant 245 µA RMS sinusoidal current at 50 kHz. The system measures resistance within a 0–1500 Ω range and reactance within 0–500 Ω, with a tolerance below 2%. Calibration using a precision resistor–capacitor circuit showed errors < 1 Ω for resistance and < 2% for reactance. All L-BIA assessments used 50-mm diameter Ag/AgCl adhesive electrodes (Ambu® WhiteSensor Ref. CMM/1-00-S/30, Brussels, Belgium) with intrinsic values of R = 27 Ω and Xc = 0.25 Ω.

### Data analysis

The primary outcome of the study was the comparison of postoperative quadriceps muscle recovery between the ultracongruent and medial-pivot groups, assessed longitudinally by serial Xc measurements obtained by localized bioelectrical impedance analysis (L-BIA). Analyses of resistance (R), clinical scores, radiographic parameters, and other secondary endpoints were considered exploratory and interpreted accordingly.

Data normality was assessed using the Shapiro–Wilk test and homogeneity of variances with Levene’s test. Normally distributed variables are presented as mean ± standard deviation (SD) with 95% confidence intervals (CI), whereas non-normally distributed variables are expressed as median and interquartile range (IQR). Categorical variables are reported as frequencies and percentages.

Temporal changes in the operated limb (preoperative, 6 months, and 12 months postoperatively) were analyzed using repeated-measures ANOVA with Bonferroni-adjusted post hoc tests, or the Friedman test with pairwise comparisons for non-normally distributed data. Comparisons between affected and unaffected limbs were performed using paired-samples t tests or Wilcoxon signed-rank tests, while comparisons between TKA designs used Student’s t test or Mann–Whitney U test as appropriate. No additional adjustment for multiple comparisons was applied to between-group or other exploratory analyses. Therefore, *p*-values from these analyses should be interpreted as exploratory rather than confirmatory.

To account for interindividual variability in bioelectrical reactance (Xc), percentage change (%Δ) values were calculated [46]. Because baseline differences may influence percentage-based comparisons, analysis of covariance (ANCOVA) was used, when appropriate, to adjust for baseline values and isolate the effect of implant design on postoperative outcomes. Before interpretation of the ANCOVA models, model assumptions were evaluated. Homogeneity of regression slopes was assessed by testing the interaction between TKA type and the baseline covariate in each ANCOVA model. Homogeneity of variances was evaluated using Levene’s test, and residual distributions were assessed using Q–Q plots and the Shapiro–Wilk test. Effect sizes were reported as partial eta-squared (η^2^p). Given the limited sample size, ANCOVA analyses were considered exploratory rather than confirmatory evidence.

Statistical analyses were performed using a complete-case approach, including only participants who underwent the allocated intervention, completed study follow-up, and had complete primary outcome data available for the primary analysis. Participants with missing primary outcome data were excluded from the primary analyses, as shown in Fig. 1. No imputation of missing outcome data was performed. Given the small sample size and the limited information available to support reliable imputation of missing longitudinal L-BIA outcomes, an intention-to-treat analysis including all randomized participants was not performed.

The study was designed as a superiority trial to evaluate whether differences in postoperative muscle recovery existed between implant designs. No equivalence or non-inferiority margins were prespecified; therefore, the absence of statistically significant differences should not be interpreted as evidence of equivalent implant performance. All analyses were two-tailed, with statistical significance set at *p* < 0.05. Statistical analyses were performed using IBM SPSS Statistics version 30.0 (IBM Corp., Armonk, NY, USA).

## Results

### Demographics variables

Demographic variables are shown in Table 1. No statistically significant between-group differences were observed in demographic or preoperative clinical characteristics; however, some baseline imbalances were present, particularly in sex distribution, height, and weight. Preoperative whole-body bioelectrical impedance measurements (R, Xc, PhA, R/H, and Xc/H) suggested a similar baseline global bioelectrical profile between groups (Table 1).

**Table 1:** Demographic data

		**MP (*n* = 15)**	**UC (*n* = 15)**	***p-*value**^**T**^ **Cohen’s d**
**Age (y.o)**		71.1 ± 7.8 (66.8–75.4)	70.1 ± 6.2 (66.7–73.5)	0.68 0.15
**Weight (kg)**		77.7 ± 12.7 (70.7–84.8)	84.0 ± 10.6 (78.1–89.8)	0.15 − 0.54
**Height (m)**		1.59 ± 0.1 (1.55–1.62)	1.63 ± 0.1 (1.59–1.69)	0.08 − 0.70
**BMI (kg/m**^**2**^**)**		31.0 ± 4.7 (28.5–33.6)	31.4 ± 4.9 (28.8–34.2)	0.81 − 0.09
**Whole-Body Bioimpedance**	R (Ω)	497.3 ± 102.4 (440.6–554.0)	514.1 ± 70.7 (474.9–553.2)	0.60 − 0.20
	Xc (Ω)	54.6 ± 16.1 (45.7–63.5)	48.1 ± 6.1 (44.7–51.5)	0.15 0.54
	PhA (º)	6.5 ± 2.5 (5.1–7.9)	5.4 ± 0.5 (5.1–5.6)	0.10 0.64
	R/H (Ω/m)	313.8 ± 67.0 (276.7–350.9)	314.6 ± 50.5 (286.6–342.6)	0.97 − 0.01
	Xc/H (Ω/m)	34.5 ± 10.6 (28.7–40.4)	25.3 ± 10.8 (19.3–31.3)	0.05 0.80
				***p-*value**^**χ2**^ **φ/V**
**Sex, *n* (%)**	Women	12 (80.0)	8 (53.3)	0.12 0.28
	Men	3 (20.0)	7 (46.7)	
**Side, *n* (%)**	Right	7 (46.7)	4 (26.7)	0.26 0.21
	Left	8 (53.3)	11 (73.3)	
**ASA, N (%)**	I	0 (0.0)	1 (6.7)	0.36 0.26
	II	12 (80.0)	13 (86.7)	
	III	3 (20.0)	1 (6.7)	
	IV	0 (0.0)	0 (0.0)	

Baseline demographic, clinical characteristics and basal body bioelectrical measurements of patients according to prosthesis design (medial pivot (MP) vs ultracongruent (UC)). Data are expressed as mean ± standard deviation (SD) and 95%CI or absolute value and percentage (%). *p*-value^T^ correspond to comparisons between groups calculated by Student’s t-test for continuous variables and Chi-square test for categorical variables (*p*-value^χ2^). Effect size for the Student’s t-test was expressed as Cohen’s d, whereas effect size for chi-square tests was quantified using Phi (φ) for 2 × 2 contingency tables and Cramer’s V for tables larger than 2 × 2

Legend: Whole-body = preoperative whole-body bioelectrical impedance analysis; R = resistance; Xc = reactance; PhA = phase angle; R/H = resistance normalized to height; Xc/H = reactance normalized to height

Although moderate standardized effect sizes were observed for height (d = 0.70) and weight (d = 0.54), BMI was well balanced between groups (d = 0.09).

### L-BIA parameters in operated leg

R values remained stable across the preoperative, 6-month, and 12-month assessments, with no differences between MP and UC designs at any time point (Table 2).

**Table 2:** Quadriceps muscle groups L-BIA parameters pre- and post- TKA surgery divided by TKA prosthesis design

Muscle group	LBIA	TKA type	Pre TKA	Post TKA 6 m	Post TKA 12 m	*p*-value^F^ Kendall’s W	*p*-value^F(1–2)^ r	*p*-value^F(2–3)^ r	*p*-value^F(1–3)^ r
**Rectus Femoris**	R (Ω)	MP	119.0 (50.7) (76.3–167.7)	106.0 (37.6) (62.0–146.2)	120.0 (33.2) (57.1–186.2)	0.09 0.10	0.07 0.39	0.06 0.43	0.84 0.03
		UC	123.0 (53.9) (67.5–185.8)	107.0 (31.5) (64.2–165.2)	113.3 (41.4) (67.1–171.6)	0.63 0.04	0.38 0.25	0.86 0.05	0.48 0.20
		*p*-value^MWU^ *r*	0.96 − 0.01	0.74 0.08	0.84 − 0.05				
	Xc (Ω)	MP	7.0 (2.0) (4.5–15.6)	6.2 (2.1) (4.4–15.0)	7.9 (2.1) (4.8–16.3)	< 0.01* 0.54	< 0.01* 0.78	< 0.01* 0.80	0.05 0.60
		UC	8.0 (1.8) (4.5–10.6)	7.4 (3.5) (3.5–10.8)	8.0 (2.2) (5.7–10.9)	0.06 0.22	0.21 0.36	0.02* 0.71	0.21 0.36
		*p*-value^MWU^ *r*	0.26 0.24	0.17 0.30	0.45 0.15				
**Vastus Medialis**	R (Ω)	MP	125.0 (55.5) (72.5–175.0)	129.0 (53.7) (70.8–208.3)	124.7 (40.0) (84.6–193.5)	0.63 0.03	0.86 0.05	0.38 0.25	0.48 0.20
		UC	131.0 (53.9) (64.0–202.2)	127.0 (41.6) (65.4–165.3)	128.2 (47.5) (62.0–186.8)	0.29 0.10	0.86 0.05	0.21 0.36	0.15 0.41
		*p*-value^MWU^ *r*	0.90 − 0.03	0.78 − 0.07	0.65 − 0.10				
	Xc (Ω)	MP	7.3 (2.4) (5.7–17.7)	7.0 (1.5) (4.5–18.2)	7.6 (3.8) (4.9–20.4)	< 0.01* 0.45	0.18 0.38	< 0.01* 0.80	0.05 0.76
		UC	8.1 (1.7) (5.0–13.8)	7.8 (2.9) (3.3–14.5)	8.1 (3.1) (5.7–15.0)	0.07 0.20	0.57 0.16	0.03* 0.65	0.10 0.48
		*p*-value^MWU^ *r*	0.32 0.21	0.72 0.08	0.69 0.09				
**Vastus Lateralis**	R (Ω)	MP	116.0 (46.7) (68.4–168.4)	120.0 (38.3) (63.5–166.2)	118.8 (35.3) (60.8–206.7)	0.42 0.07	0.72 0.10	0.37 0.25	0.21 0.35
		UC	112.0 (50.5) (68.7–184.1)	112.0 (34.5) (58.6–152.8)	120.4 (37.7) (54.1–175.6)	0.42 0.07	0.21 0.35	0.72 0.10	0.37 0.25
		*p*-value^MWU^ *r*	0.81 − 0.06	0.54 − 0.14	0.81 − 0.06				
	Xc (Ω)	MP	8.0 (2.5) (5.8–15.2)	6.9 (3.9) (4.6–23.0)	7.8 (4.9) (5.3–25.8)	0.03* 0.25	0.02* 0.68	0.02* 0.68	0.98 0.00
		UC	9.5 (3.2) (5.8–14.1)	8.3 (2.7) (4.5–10.5)	9.1 (2.3) (4.7–12.6)	0.10 0.18	0.05 0.60	0.16 0.46	0.38 0.14
		*p*-value^MWU^ *r*	0.32 0.22	0.56 0.13	0.51 0.15				

MP: medial pivot; UC: ultracongruent. Ω, Ohm: unit of the International System used to quantify resistance and impedance

Evolution of L-BIA parameters (R and Xc) in the operated leg over time according to implant design. Values are expressed as median and IQR (interquartile range), and minimum–maximum. Comparisons were performed within each group across time points (1: pre-TKA, 2: post-TKA 6 months, and 3: post-TKA 12 months) using repeated-measures Friedman test (*p*-value^F(1–2)^; *p*-value^F(1–3)^, *p*-value^F(2–3)^) and between implant designs using Mann–Whitney U-Test (*p-*value^MWU^). Effect size was expressed as Wilcoxon r (rank-biserial correlation, *r*) for both statistical tests

^*^ indicates statistical significance (*p* < 0.05)

Reactance (Xc) showed modest group differences. In the MP group, a marked decrease was observed at 6 months across all muscle groups, followed by a significant recovery at 12 months, with values returning to or exceeding baseline. The UC group showed changes in the same direction, although these were a less pronounced pattern that did not consistently reach statistical significance (Table 2). No intergroup differences were detected at any time point (Table 2).

Changes in resistance (ΔR) remained stable over time without significant temporal or between-group differences. In contrast, changes in reactance (ΔXc) showed a transient reduction at 6 months followed by substantial recovery at 12 months in all muscles, more evident in the MP group than in the UC group (Table 3). Nevertheless, no significant differences between implant designs were observed for any muscle or time point (Table 3).

**Table 3:** Percentage of change in L-BIA quadriceps muscle groups repeated measurements divided by TKA prosthesis designs

Muscle group	LBIA	TKA type	%Δ 2–1	%Δ 3–1	*p-*value^W^ *r*
**Rectus Femoris**	R (Ω)	MP	− 11.7 (17.9) (− 35.6 to 20.2)	1.7 (23.4) (− 36.5 to 27.3)	0.07 − 0.53
		UC	− 4.9 (17.3) (− 36.0 to 29.7)	− 1.6 (21.9) (− 31.1 to 37.3)	0.72 − 0.12
		*p*-value^MWU^ *r*	0.49 0.16	0.81 − 0.05	
	Xc (Ω)	MP	− 11.4 (20.0) (− 38.7 to 55.0)	6.1 (22.2) (− 22.7 to 75.6)	< 0.01* − 0.98
		UC	− 7.5 (21.1) (− 24.3 to 67.2)	6.5 (28.8) (− 17.4 to 70.3)	0.08 − 0.54
		*p*-value^MWU^ *r*	0.31 0.23	0.97 − 0.01	
**Vastus Medialis**	R (Ω)	MP	2.5 (22.7) (− 23.2 to 39.6)	1.8 (15.1) (− 25.8 to 59.2)	0.28 − 0.33
		UC	0.3 (15.9) (− 26.0 to 15.6)	− 3.1 (20.2) (− 34.1 to 41.8)	0.76 0.10
		*p*-value^MWU^ *r*	0.74 − 0.08	0.22 − 0.27	
	Xc (Ω)	MP	− 7.7 (33.6) (− 37.7 to 29.8)	13.3 (25.3) (− 36.4 to 45.6)	< 0.01* − 0.82
		UC	− 16.7 (31.0) (− 40.0 to 44.0)	8.7 (19.3) (− 42.9 to 43.0)	0.06 − 0.57
		*p*-value^MWU^ *r*	0.54 − 0.13	0.68 − 0.10	
**Vastus Lateralis**	R (Ω)	MP	0.1 (17.4) (− 31.2 to 36.3)	2.4 (15.4) (− 35.2 to 25.7)	0.45 − 0.23
		UC	− 4.8 (23.1) (− 30.8 to 47.4)	− 0.6 (22.9) (− 26.6 to 41.6)	0.85 − 0.07
		*p*-value^MWU^ *r*	0.60 − 0.12	0.46 − 0.16	
	Xc (Ω)	MP	− 8.0 (18.7) (− 39.5 to 33.3)	− 3.8 (34.6) (− 30.3 to 98.4)	< 0.01* − 0.75
		UC	− 14.8 (18.8) (− 40.0 to 17.1)	− 0.8 (20.8) (− 25.5 to 11.0)	0.09 − 0.47
		*p*-value^MWU^ *r*	0.51 − 0.15	0.41 − 0.18	

MP: medial pivot; UC: ultracongruent; Ω, Ohm: unit of the International System used to quantify resistance and impedance. %Δ2–1 = post TKA 6 m—pre TKA; %Δ3–1 = post TKA 12 m—pre TKA. Percentage change in resistance (R) and reactance (Xc) post-TKA. Values are expressed as median and IQR (interquartile range), and minimum–maximum. Comparisons were performed within each group using Wilcoxon Rank Test (*p-*value^W^) and between implant designs using Mann Whitney U-Test (*p-*value^MWU^). Effect size was expressed as Wilcoxon r (rank-biserial correlation, *r*) for both statistical tests

^*^ indicates statistical significance (*p* < 0.05)

Analysis of covariance (ANCOVA) was performed to evaluate whether implant design influenced 12-month postoperative Xc after adjustment for baseline Xc. Baseline Xc was associated with 12-month postoperative Xc, whereas implant design had no significant effect (Table 4).

**Table 4:** Analysis of covariance (ANCOVA) for muscle reactance (Xc) at 12 months, adjusted for baseline values

Muscle group	Source	SS	Df	Fisher	*p*-value	Adjusted 12-month Xc
**Rectus femoris (RF)**	Xc_pre TKA (Ω)	83.92	1	33.81	< 0.001*	
	Type TKA	0.13	1	0.05	0.820	MP: 8.44 (7.61–9.28) UC: 8.31 (7.48–9.15)
**Vastus medialis (VM)**	Xc_pre TKA (Ω)	230.54	1	61.47	< 0.001*	
	Type TKA	2.15	1	0.57	0.455	MP: 9.33 (8.30–10.36) UC: 8.80 (7.77–9.82)
**Vastus lateralis (VL)**	Xc_pre TKA (Ω)	314.56	1	66.14	< 0.001*	
	Type TKA	11.83	1	2.49	0.126	MP: 10.13 (8.97–11.30) UC: 8.87 (7.72–10.00)

Analysis of covariance (ANCOVA) evaluating the effect of implant design (MP vs UC) on bioelectrical reactance (Xc) at 12 months, adjusted for baseline (pre-TKA) Xc values. Adjusted 12-month Xc values are presented as estimated marginal means with 95% confidence intervals. The assumption of homogeneity of regression slopes was assessed by testing the interaction between baseline Xc and TKA type. No statistically significant interaction was observed for the RF (*p* = 0.149), VM (*p* = 0.06), and VL (*p* = 0.262)

Legend: SS, sum of squares; df, degrees of freedom; F, Fisher F-ratio; Xc, reactance; MP, medial pivot; UC, ultracongruent; CI, confidence interval; Ω, Ohm: unit of the International System used to quantify resistance and impedance.; pre-TKA, preoperative.

^*^ indicates statistical significance (*p* < 0.05)

In sensitivity analyses additionally adjusted for sex and BMI, baseline Xc remained positively associated with 12-month postoperative Xc across all three muscle groups, whereas implant design, sex, and BMI showed no significant associations (Supplementary Table S1).

### Interleg L-BIA parameters

No significant differences in R values were found between the affected and unaffected legs for any muscle, either pre- or post-TKA. These values were bilaterally comparable at both time points, and no differences were detected between implant designs (Table 5).

**Table 5:** L-BIA quadriceps muscle groups pre- and 12 months post-TKA surgery: comparison between affected and unaffected leg and between both types of TKA designs

			**Pre-TKA**			**Post-TKA**		
**Muscle group**	**LBIA**	**TKA type**	**Affected**	**Unaffected**	***p-*value**^**W**^ ***r***	**Affected**	**Unaffected**	***p-*value**^**W**^ ***r***
**Rectus Femoris**	R (Ω)	MP	119.0 (50.7) (76.3–167.7)	117.1 (35.3) (79.4–164.3)	0.98 − 0.02	120.0 (33.2) (57.1–186.2)	116.8 (26.1) (67.4–179.9)	0.85 − 0.07
		UC	123.0 (53.9) (67.5–185.8)	126.6 (54.5) (61.0–168.1)	0.23 0.38	113.3 (41.4) (67.1–171.6)	114.0 (45.6) (56.8–171.9)	0.36 − 0.28
		*p*-value^MWU^ *r*	0.97 − 0.01	0.77 − 0.07		0.84 − 0.05	0.95 − 0.02	
	Xc (Ω)	MP	7.0 (2.0) (4.5–15.6)	7.0 (2.8) (5.2–12.5)	0.20 − 0.41	7.9 (2.1) (4.8–16.3)	7.2 (2.9) (5.2–18.7)	0.70 − 0.10
		UC	8.0 (1.8) (4.5–10.6)	8.2 (2.3) (4.5–13.0)	0.10 − 0.48	8.0 (2.2) (5.7–10.9)	8.1 (1.6) (5.6–11.6)	0.85 − 0.05
		*p*-value^MWU^ *r*	0.26 0.24	0.26 0.24		0.99 0.00	0.27 0.24	
**Vastus Medialis**	R (Ω)	MP	125.0 (55.5) (72.5–175.0)	132.2 (50.2) (80.2–174.1)	0.68 − 0.14	124.7 (40.0) (84.6–193.5)	118.3 (31.8) (76.1–210.3)	0.87 − 0.06
		UC	131.0 (53.9) (64.0–202.2)	133.5 (53.7) (61.6–201.1)	0.63 − 0.15	128.2 (47.5) (62.0–186.8)	112.5 (64.6) (72.8–181.6)	0.42 − 0.25
		*p*-value^MWU^ *r*	0.90 − 0.03	0.90 0.03		0.65 − 0.10	0.56 − 0.13	
	Xc (Ω)	MP	7.3 (2.4) (5.7–17.7)	8.1 (1.9) (6.3–15.4)	0.15 − 0.43	7.6 (3.8) (4.9–20.4)	8.4 (5.0) (4.3–25.0)	0.15 − 0.40
		UC	8.1 (1.7) (5.0–13.8)	8.5 (3.0) (4.8–16.2)	0.33 − 0.30	8.1 (3.1) (6.0–15.7)	9.2 (2.4) (5.1–13.6)	0.50 − 0.18
		*p*-value^MWU^ *r*	0.33 0.21	0.60 0.12		0.81 0.09	0.98 0.01	
**Vastus Lateralis**	R (Ω)	MP	116.0 (46.7) (68.4–168.4)	119.6 (51.3) (70.3–175.5)	0.85 − 0.07	118.8 (35.3) (60.8–206.7)	122.9 (33.1) (61.6–185.0)	0.81 0.09
		UC	112.0 (50.5) (68.7–184.1)	110.8 (36.5) (57.2–174.5)	0.73 0.11	120.4 (37.7) (54.1–175.6)	122.1 (33.1) (57.4–180.6)	0.95 − 0.01
		*p*-value^MWU^ *r*	0.81 − 0.06	0.65 − 0.10		0.81 − 0.06	0.38 − 0.20	
	Xc (Ω)	MP	8.0 (2.5) (5.8–15.2)	8.7 (2.4) (6.7–17.3)	0.10 − 0.55	7.8 (4.9) (5.3–25.8)	8.2 (7.1) (6.4–25.4)	0.15 − 0.39
		UC	9.5 (3.2) (5.8–14.1)	10.0 (2.1) (5.4–13.7)	0.13 − 0.45	9.1 (2.3) (4.7–12.6)	11.1 (2.5) (4.8–12.8)	0.05 − 0.60
		*p*-value^MWU^ *r*	0.32 0.22	0.19 0.28		0.53 0.23	0.49 0.15	

Comparison of L-BIA parameters (R and Xc) between affected and unaffected leg before and after TKA. Values are expressed as median and IQR (interquartile range), minimum–maximum. Comparisons were performed between the affected and unaffected leg within each implant design (Medial Pivot and Ultracongruent) using Wilcoxon Rank Test (*p*-value^W^) both preoperatively and postoperatively, as well as between designs by Mann Whitney U-Test (*p*-value^MWU^). Effect size was expressed as Wilcoxon r (rank-biserial correlation, *r*) for both statistical tests

Similarly, Xc values showed no significant differences preoperatively or postoperatively in any muscle. Both implant designs demonstrated a parallel evolution of Xc in the affected and unaffected leg (Table 5).

### Radiographic analysis

No significant differences were observed between medial-pivot (MP) and ultracongruent (UC) prostheses with respect to joint line height, patellar height, posterior condylar offset ratio, tibial slope, or mechanical/anatomical alignment, either preoperatively or postoperatively. Similarly, the radiographic changes observed after TKA did not differ significantly between groups. (Table 6).

**Table 6:** Comparison of pre- and postoperative radiographic and clinical measurements between medial pivot and ultracongruent prostheses

				**MP** **(*n* = 15)**	**UC** **(*n* = 15)**	***p-*value**^**T**^ **Cohen’s d**
**Radiographic measurements**	Joint line (mm) *(Peroneal reference)*	Pre-TKA		15.20 ± 3.93 (13.0–17.4)	16.16 ± 2.52 (14.76–17.56)	0.44 − 0.29
		Post-TKA		15.28 ± 3.14 (13.5–17.0)	15.33 ± 2.96 (13.69–16.97)	0.96 − 0.02
		Δ post–pre		0.07 ± 3.33 (− 1.77 to 1.91)	− 0.83 ± 3.47 (− 2.75 to 1.09)	0.47 0.27
		*p*-value^PTT^ Cohen’s d		0.94 − 0.02	0.37 0.24	
	Joint line (mm) *(Adductor tubercle)*	Pre-TKA		42.50 ± 4.18 (40.20–44.80)	46.20 ± 5.21 (43.40–49.10)	0.04* − 0.79
		Post-TKA		42.90 ± 3.67 (40.90–45.00)	46.10 ± 4.32 (43.70–48.50)	0.04* − 0.79
		Δ post–pre		0.43 ± 1.42 (− 0.36 to 1.21)	− 0.12 ± 2.07 (− 1.27 to 1.03)	0.41 0.31
		*p*-value^PTT^ Cohen’s d		0.26 − 0.30	0.83 0.06	
	Patellar Height (mm) *(Insall-Salvati)*	Pre-TKA		1.08 ± 0.13 (1.00–1.15)	1.06 ± 0.13 (0.99–1.13)	0.75 0.12
		Post-TKA		1.12 ± 0.15 (1.03–1.20)	1.11 ± 0.11 (1.05–1.17)	0.93 0.33
		Δ post–pre		0.04 ± 0.06 (0.00–0.07)	0.05 ± 0.08 (0.00–0.09)	0.68 − 0.15
		*p*-value^PTT^ Cohen’s d		0.04* − 0.60	0.03* − 0.65	
	Patellar Height (mm) *(Caton-Deschamps)*	Pre-TKA		1.10 ± 0.22 (0.98–1.22)	1.14 ± 0.20 (1.03–1.26)	0.53 − 0.24
		Post-TKA		1.08 ± 0.19 (0.97–1.13)	1.13 ± 0.15 (1.04–1.21)	0.11 − 0.60
		Δ post–pre		− 0.07 ± 0.19 (− 0.18 to 0.03)	− 0.02 ± 0.10 (− 0.07 to 0.04)	0.34 − 0.36
		*p*-value^PTT^ Cohen’s d		0.46 0.38	0.50 0.18	
	Ratio of Posterior condylar offset	Pre-TKA		0.41 ± 0.04 (0.39–0.43)	0.42 ± 0.04 (0.40–0.44)	0.31 − 0.38
		Post-TKA		0.43 ± 0.04 (0.40–0.45)	0.43 ± 0.05 (0.40–0.46)	0.82 − 0.08
		Δ post–pre		0.02 ± 0.02 (0.00–0.03)	0.01 ± 0.04 (− 0.01 to 0.03)	0.40 0.32
		*p*-value^PTT^ Cohen’s d		< 0.01* − 0.82	0.38 − 0.24	
	Tibial slope (º)	Pre-TKA		9.70 ± 3.44 (7.80–11.60)	10.33 ± 4.95 (7.59–13.07)	0.70 − 0.14
		Post-TKA		5.20 ± 2.11 (4.03–6.35)	5.07 ± 2.61 (3.62–6.51)	0.89 0.05
		Δ post–pre		− 4.53 ± 4.23 (− 6.88 to − 2.18)	− 5.27 ± 5.05 (− 8.06 to − 2.47)	0.67 0.16
		*p*-value^PTT^ Cohen’s d		< 0.01* 1.07	< 0.01* 1.04	
	Mechanical axis (º)	Pre-TKA		172.68 ± 2.95 (170.80–174.56)	172.40 ± 4.37 (169.76–175.04)	0.86 0.07
		Post-TKA		177.14 ± 1.89 (176.00–178.28)	175.97 ± 2.17 (174.66–177.29)	0.16 0.57
		Δ post–pre		4.77 ± 4.08 (2.03–7.51)	3.99 ± 3.81 (1.44–6.55)	0.65 0.20
		*p*-value^PTT^ Cohen’s d		< 0.01* − 1.17	< 0.01* − 1.05	
	Anatomical axis (°)	Pre-TKA		170.55 ± 6.40 (166.48–174.62)	176.64 ± 2.04 (175.41–177.87)	0.01* − 1.31
		Post-TKA		173.57 ± 3.59 (171.40–175.74)	172.36 ± 9.92 (166.37–178.36)	0.68 0.16
		Δ post–pre		2.35 ± 8.30 ((− 3.22)–7.90)	− 3.89 ± 9.78 ((− 10.46)–2.69)	0.12 0.69
		*p*-value^PTT^ Cohen’s d		0.37 − 0.28	0.22 0.40	
**Clinical measurements**	Range of motion (º)	Pre-TKA		113.67 ± 8.10 (109.17–118.16)	111.34 ± 14.45 (103.33–119.34)	0.59 0.20
		Post-TKA		108.34 ± 9.20 (103.24–113.43)	105.67 ± 12.52 (98.74–112.60)	0.51 0.24
		Δ post–pre		− 5.30 ± 11.30 ((− 11.56)–0.90)	− 5.67 ± 16.78 ((− 14.96)–3.63)	0.95 0.02
		*p*-value^PTT^ Cohen’s d		0.09 0.47	0.21 0.34	

Radiographic parameters and clinical measurements pre and post TKA in both prosthesis designs (medial pivot (MP) and ultracongruent (UC)). Δ post–pre values represent the difference between postoperative and preoperative measurements. Data are presented as mean ± standard deviation (SD) and 95%CI. *p-*values^PTT^ reflect comparisons intragroup (Paired-T test), while *p-*values^T^ correspond to comparisons between groups (Student’s T-test). Effect size was represented by Cohen’s d

^*^ indicates statistical significance (*p* < 0.05)

JL measured using the fibular head reference showed no differences between groups pre- or postoperatively, with minimal positional changes, indicating good joint line preservation. When measured from the adductor tubercle, UC showed higher JL values than MP at baseline and postoperatively (*p* = 0.04), but the change over time did not differ between groups, suggesting baseline anatomical variation rather than a surgical effect.

Patellar height did not differ between groups using either the Insall–Salvati ratio or the Caton–Deschamps index. Both designs showed a small but significant postoperative increase in Insall–Salvati values (MP *p* = 0.04; UC *p* = 0.03), with negligible intergroup differences.

PCOR values were similar between groups pre- and postoperatively. A small increase was observed within the MP group (*p* < 0.01), while no change occurred in UC (*p* = 0.38); however, ANCOVA adjusted for baseline values showed no significant between-group differences (F = 0.60, *p* = 0.446).

No statistically significant between-group difference in tibial slope was observed at baseline, and tibial slope decreased significantly after TKA in both groups (*p* < 0.01), with no statistically significant difference in postoperative changes between implant designs.

Postoperative ROM was similar between groups (108.3 ± 9.2° in the MP group vs. 105.7 ± 12.5° in the UC group; *p* = 0.51). After adjustment for baseline ROM using ANCOVA, no significant effect of implant design was observed (*p* = 0.587). The estimated marginal mean postoperative ROM was 108° (95% CI 102–114) for the MP group and 106° (95% CI 100–112) for the UC group, with an adjusted between-group difference of 2°, which was neither statistically nor clinically significant. The assumption of homogeneity of regression slopes was met, as the interaction between baseline ROM and TKA type was not statistically significant (*p* = 0.962).

Mechanical axis alignment improved significantly after TKA in both groups (*p* < 0.01) with no differences between groups. Although a baseline difference in anatomical axis was observed (UC > MP, *p* = 0.01), it disappeared postoperatively, indicating effective correction in both designs.

### Clinical evaluation

Both implant designs showed a significant improvement in total WOMAC scores over time (*p* < 0.01), with the greatest improvement occurring during the first 6 months. Pain, stiffness, and functional subscale scores also improved significantly in both groups (*p* < 0.01), with the largest reductions observed within the first 6 months. No intergroup differences were detected at any time point. However, UC showed a slight additional improvement between 6 and 12 months in the total and function subscales, although this did not reach statistical significance (Table 7).

**Table 7:** Comparison of WOMAC total and subscale scores before surgery and at 6 and 12 months according to prosthesis design

WOMAC	TKA	Pre-TKA	Post-TKA 6 m	Post-TKA 12 m	*p*-value^F^ Kendall’s W	*p*-value^F(1–2)^ *r*	*p*-value^F(2–3)^ *r*	*p*-value^F(1–3)^ *r*
**Total**	MP	55 (13.5) (27–72)	21 (14.5) (5–45)	20 (17.5) (3–41)	< 0.01* 0.65	< 0.01* 1.00	0.71 0.14	< 0.01* 1.00
	UC	45 (20) (24–69)	18 (10) (4–42)	11.5 (13.8) (0–27)	< 0.01* 0.74	< 0.01* 1.00	0.03* 0.76	< 0.01* 1.00
	*p*-value^MWU^ *r*	0.18 − 0.29	0.47 − 0.16	0.18 − 0.30				
**Pain**	MP	11 (4) (8–17)	4 (3.8) (0–11)	3 (2) (0–9)	< 0.01* 0.56	< 0.01* 0.98	0.16 0.36	< 0.01* 1.00
	UC	10 (3.5) (6–14)	3 (5.5) (0–9)	2 (2.8) (0–6)	< 0.01* 0.75	< 0.01* 1.00	0.21 0.33	< 0.01* 1.00
	*p*-value^MWU^ *r*	0.08 − 0.37	0.71 − 0.09	0.32 − 0.22				
**Stiffness**	MP	4 (1.5) (2–6)	2 (2.8) (0–6)	1.5 (1.8) (0–4)	< 0.01* 0.38	0.05 0.75	0.02* 0.72	< 0.01* 0.97
	UC	3 (3) (0–7)	2 (2) (0–4)	1 (2) (0–3)	< 0.01* 0.37	0.07 0.56	0.03* 0.73	< 0.01* 0.90
	*p*-value^MWU^ *r*	0.22 − 0.25	0.58 − 0.12	0.40 − 0.18				
**Function**	MP	39 (9.5) (19–52)	14 (15) (1–31)	14 (16.5) (3–31)	< 0.01* 0.66	< 0.01* 1.00	0.26 − 0.05	< 0.01* 1.00
	UC	37 (12.5) (14–55)	12 (8) (3–29)	9.5 (10.3) (0–20)	< 0.01* 0.77	< 0.01* 1.00	0.05 0.73	< 0.01* 1.00
	*p*-value^MWU^ *r*	0.49 − 0.15	0.84 − 0.05	0.18 − 0.30				

MP: medial pivot; UC: ultracongruent. Values are presented as median and IQR, minimum–maximum

Comparisons of WOMAC score and subgroups across time points (1: pre-TKA, 2: post-TKA 6 months, and 3: post-TKA 12 months) within each group were calculated using repeated-measures Friedman tests (*p*-value^F(1–2)^; *p*-value^F(1–3)^, *p*-value^F(2–3)^) and between TKA designs with Mann Whitney U-Test (*p*-value^MWU^). Effect size for Friedman’s test was expressed as Kendall’s W, while for Mann Whitney U-Test was Wilcoxon r (r)

^*^ indicates statistical significance (*p* < 0.05)

Both implant designs showed a significant improvement in total KSS scores and subscores (knee and function) over time (*p* < 0.01), with the most pronounced improvement occurring within the first 6 months. Between 6 and 12 months, scores remained stable in both groups, with no significant additional gains. No significant differences in clinical or functional outcomes were observed between implant designs at any time point during the first postoperative year. (Table 8).

**Table 8:** Comparison of KSS total and subscale scores before surgery and at 6 and 12 months according to prosthesis design

KSS	TKA	Pre-TKA	Post-TKA 6 m	Post-TKA 12 m	*p*-value^F^ Kendall’s W	*p*-value^F(1–2)^ *r*	*p*-value^F(2–3)^ *r*	*p*-value^F(1–3)^ *r*
**Total**	MP	98 (37) (67–137)	162 (25.5) (130–197)	171 (26) (70–197)**	< 0.01* 0.68	< 0.01* − 1.00	0.53 − 0.29	< 0.01* − 0.95
	UC	109 (19) (89–137)	169 (27) (89–192)	168.5 (32.5) (132–199)	< 0.01* 0.72	< 0.01* − 0.98	0.15 − 0.38	< 0.01* − 1.00
	*p*-value^MWU^ *r*	0.09 0.37	0.77 0.06	0.76 0.07				
**Knee**	MP	54 (15) (22–68)	87 (19) (65–99)	91 (13) (45–100)	< 0.01* 0.61	< 0.01* − 0.98	0.26 − 0.22	< 0.01* − 0.93
	UC	59 (7.5) (44–67)	87 (9.5) (44–94)	92.5 (11.5) (71–99)	< 0.01* 0.70	< 0.01* − 0.95	0.05 − 0.40	< 0.01* − 1.00
	*p*-value^MWU^ *r*	0.59 0.12	0.79 − 0.06	0.65 0.10				
**Function**	MP	50 (15) (15–70)	80 (20) (65–100)	90 (10) (25–100)	< 0.01* 0.65	< 0.01* − 1.00	0.65 − 0.10	< 0.01* − 0.98
	UC	55 (15.5) (30–70)	80 (25) (45–100)	90 (30) (60–100)	< 0.01* 0.74	< 0.01* − 1.00	0.31 − 0.18	< 0.01* − 1.00
	*p*-value^MWU^ *r*	0.16 0.31	0.98 0.01	0.66 0.10				

MP: medial pivot; UC: ultracongruent. Values are presented as median and IQR, minimum–maximum

Comparisons of KSS score and subgroups across time points (1: pre-TKA, 2: post-TKA 6 months, and 3: post-TKA 12 months) within each group using repeated-measures Friedman tests (*p*-value^F(1–2)^; *p*-value^F(1–3)^, *p*-value^F(2–3)^) and between TKA designs with Mann Whitney U-Test (*p*-value^MWU^). Effect size for Friedman’s test was expressed as Kendall’s W, while for Mann Whitney U-Test was Wilcoxon r *(r*)

^*^ indicates statistical significance (*p* < 0.05)

^**^The minimum KSS total score of 70 in the MP group at 12 months corresponded to a single outlier; therefore, median and IQR were reported to better represent the group distribution

## Discussion

The main finding of this study is that no significant between-group differences were observed in clinical outcomes or quadriceps bioelectrical trajectories following medial-pivot and ultracongruent TKA. Preoperative reactance was consistently associated with postoperative Xc values, whereas implant design showed no significant association with postoperative bioelectrical muscle status during the first postoperative year. Importantly, L-BIA-derived Xc should be interpreted as a bioelectrical surrogate of muscle status rather than as a direct measure of muscle strength, morphology, or functional recovery.

To our knowledge, this is the first study to directly compare medial pivot and ultracongruent designs while evaluating postoperative quadriceps muscle adaptation using localized bioelectrical impedance analysis (L-BIA), an objective technique for muscle assessment. Previous studies comparing implant designs have mainly focused on clinical, patient-reported, or radiographic outcomes. In contrast, the present study offers a physiological perspective on postoperative recovery by assessing muscle tissue behavior, highlighting the potential value of L-BIA for monitoring quadriceps muscle recovery after TKA.

### Quadriceps atrophy and preoperative asymmetry

Quadriceps weakness and atrophy are well-recognized sequelae of knee osteoarthritis [10–12]. Although these alterations are usually more pronounced in the painful limb, bilateral deficits have also been reported compared with healthy individuals. [10–12, 47, 48]. The quadriceps is not uniformly affected across its components: VM and VL are often more compromised than RF, likely due to their greater role in knee stabilization and higher activation during functional tasks such as standing and gait [15, 30].

In the present study, preoperative Xc values were lower in the affected limb, particularly in VM and VL, suggesting pre-existing muscle impairment. Although not statistically significant, the moderate effect sizes indicate a potentially relevant asymmetry between limbs. Quadriceps atrophy has been associated with structural and compositional changes, including increased intramuscular fatty infiltration [13–15]. Although these features were not directly assessed in the present study, they may influence the electrical properties measured by L-BIA and could partly explain the lower Xc values observed in the affected limb.

### Postoperative changes and evolution of quadriceps muscles

TKA inevitably impairs quadriceps muscle status due to surgical trauma and postoperative inflammatory responses [10–12]. At the cellular level, TKA has been associated with significant muscle fiber atrophy and contractile dysfunction, particularly affecting type II fibers (MHC II) [15]. Moreover, the extent of quadriceps involvement may also vary according to prosthetic design, as different implants present distinct kinematics, stability profiles, and load transmission patterns that may influence postoperative muscle activation and recovery.

In our study, resistance values showed only minimal and transient changes over time, a finding that may be related to postoperative changes in tissue hydration. No differences were observed between prosthetic designs. In contrast, reactance showed a distinct temporal pattern, with an early decline up to 6 months followed by progressive recovery toward baseline values at 12 months. These findings suggest temporal changes in the electrical properties of quadriceps muscle tissue. Although postoperative edema and tissue remodeling may influence localized bioimpedance measurements, assessments were performed over the quadriceps muscle bellies rather than directly over the knee joint, potentially reducing the influence of periarticular edema and scar tissue. [29]. Nevertheless, the contribution of local inflammation, scar tissue formation, and other postoperative tissue changes cannot be completely excluded.

Over the first 6 months, Xc values remained reduced across all muscle groups, indicating persistent alterations in muscle tissue bioelectrical properties. These findings are consistent with previous studies reporting prolonged quadriceps weakness and muscle loss during the early postoperative period after TKA [10–12, 48–50]. However, the timing of recovery onset remains uncertain, as reports vary from a few weeks after surgery to beyond six months postoperatively.

Over the 12-month follow-up, numerical differences in Xc changes were observed between the MP and UC groups; however, these differences did not reach statistical significance. The clinical relevance of these observations remains uncertain, and no conclusions can be drawn regarding a potential influence of implant design on postoperative muscle tissue characteristics assessed by L-BIA. Given the lack of direct measures of muscle morphology, strength, or activation, these findings should be interpreted with caution.

By 12 months post-TKA, Xc values tended to equalize between operated and contralateral limbs, and no significant differences were detected between implant designs. This finding suggests that L-BIA-derived tissue characteristics changed in the same direction over time in both groups.

In the ANCOVA models, preoperative reactance was significantly associated with postoperative Xc values, whereas prosthetic design had no significant effect. These findings suggest that preoperative muscle status may be relevant to postoperative quadriceps bioelectrical trajectories, although the present exploratory analyses do not establish a predictive or causal relationship. Previous studies have shown that preoperative quadriceps strength and muscle quality are associated with functional recovery after TKA [10–14], while greater intramuscular fatty infiltration has been associated with poorer postoperative outcomes [14, 15, 51]. In this context, chronic structural and neuromuscular alterations associated with knee osteoarthritis may constrain the extent or rate of postoperative muscle restoration. Such pre-existing alterations could theoretically impose a biological constraint on postoperative recovery, potentially limiting the extent to which subtle differences in implant geometry translate into detectable differences in quadriceps bioelectrical status within the first postoperative year. However, this interpretation remains hypothetical, as muscle morphology, composition, strength, and neuromuscular activation were not directly assessed in the present study. Future studies combining L-BIA with direct structural and functional measures of muscle status are warranted to investigate this hypothesis.

Our results suggest that the bioelectrical characteristics of quadriceps muscle tissue, as assessed by L-BIA, continue to change during the first year after TKA. In this context, localized bioimpedance analysis allowed longitudinal monitoring of changes in muscle tissue bioelectrical properties, as reflected by the evolution of reactance values throughout follow-up.

### Radiographic assessment

To explore whether implant positioning or alignment-related factors differed between prosthetic designs, radiographic parameters were compared between groups. No significant differences were found in preoperative or postoperative joint line position, patellar height, or posterior tibial slope.

Subtle differences in implant geometry may nevertheless influence knee biomechanics and quadriceps loading. Posterior tibial slope can influence knee kinematics, including femoral rollback, as well as the extensor mechanism lever arm and the mechanical demands placed on the quadriceps during knee motion [52, 53]. Although the MP and UC designs incorporate different insert geometries and kinematic principles, the postoperative posterior tibial slope was nearly identical between groups (5.2° vs 5.1°). The nearly identical postoperative alignment between groups may have limited geometry-related differences in extensor mechanics and could partly explain the absence of statistically significant between-group differences in postoperative bioelectrical muscle trajectories. However, this interpretation remains speculative, as knee kinematics and quadriceps loading were not directly assessed in the present study. Therefore, whether differences in implant geometry translate into meaningful differences in muscle loading requires further investigation using dynamic kinematic and biomechanical assessments.

Although a significant pre- to postoperative change in posterior condylar offset ratio (PCOR) was observed within the MP group, no statistically significant between-group difference in postoperative PCOR was detected. This change may reflect design-specific geometric characteristics; however, its relationship with postoperative quadriceps bioelectrical status was not directly assessed in the present study.

Both designs achieved postoperative mechanical correction, with no statistically significant between-group differences in postoperative alignment or in pre- to postoperative changes across radiographic parameters. Although a difference in the preoperative anatomical axis was observed between groups, no statistically significant between-group difference in postoperative alignment was detected, suggesting that postoperative alignment was unlikely to account for major differences in quadriceps bioelectrical trajectories.

Overall, these radiographic findings suggest that the observed patterns of quadriceps muscle adaptation were not confounded by implant positioning or alignment, supporting the interpretation that postoperative muscle recovery is primarily related to preoperative muscle status rather than prosthetic design.

### Clinical and functional outcomes

Beyond muscle adaptations, the clinical relevance of prosthetic design ultimately depends on its impact on joint balance, ROM and functional outcomes. Postoperative ROM is a key outcome after TKA. Biomechanically, MP designs may facilitate greater flexion by allowing more physiological femoral rollback, whereas UC inserts provide increased stability through higher articular conformity, potentially limiting deep flexion. However, whether these differences translate into clinically meaningful ROM differences remains controversial [4, 6–9].

In the present study, no statistically significant between-group difference in postoperative ROM was detected. Adjusted ANCOVA analysis showed no independent effect of prosthesis type after controlling for baseline ROM, suggesting that postoperative ROM is more likely influenced by patient-related and perioperative factors than by implant design. Although both groups showed a slight reduction in ROM (approximately 5°), this change was neither statistically significant nor clinically meaningful. This finding can be explained by the relatively good preoperative ROM of our cohort (> 110°), as patients with preserved baseline motion are generally less likely to achieve substantial postoperative gains. Indeed, preoperative ROM has been identified as one of the strongest predictors of postoperative ROM after TKA, with patients who have greater preoperative flexion typically maintaining, rather than improving, their postoperative range of motion [54]. The similar ROM changes observed in both groups further support that these findings were not related to implant design.

Both implant designs produced significant improvements in patient-reported outcomes. KSS (knee and function) and WOMAC scores (total, pain, and function) improved markedly within the first six months and were maintained or further enhanced at 12 months. Stiffness also improved, although to a lesser extent, consistent with previous studies reporting slower recovery in this WOMAC domain after TKA [55, 56].

Interestingly, despite the early improvement in clinical scores, reduced Xc values remained detectable at 6 months. This discrepancy may reflect the rapid postoperative reduction in pain and improvement in quality of life captured by patient-reported outcomes (PROMs), whereas L-BIA-derived parameters may reflect different aspects of postoperative tissue adaptation. Previous studies have shown that osteoarthritis and surgical trauma can be associated with prolonged changes in muscle morphology and function after TKA [15, 31]. Although muscle morphology and strength were not directly assessed in the present study, the persistence of altered bioelectrical properties at 6 months suggests that the evolution of L-BIA-derived muscle tissue characteristics may not necessarily parallel the timeline of clinical recovery after TKA.

No significant differences in WOMAC or KSS outcomes were observed between MP and UC designs at any time point. These findings suggest that factors other than implant design may contribute to postoperative patient-reported outcomes after TKA.

### Clinical implications

Overall, no significant differences in clinical, radiographic, or L-BIA outcomes were observed between implant designs during the first postoperative year. L-BIA showed temporal changes in reactance and resistance values in both groups, with no statistically significant between-group differences detected in the evolution of these bioelectrical parameters. Furthermore, adjusted analyses indicated that preoperative reactance was associated with postoperative reactance values at 12 months, whereas no significant association was observed between implant design and postoperative L-BIA outcomes. These findings indicate that no statistically significant between-group differences were detected in postoperative quadriceps bioelectrical trajectories. However, given the limited sample size and follow-up, these results should not be interpreted as sufficient to guide implant selection, and larger comparative studies are required before definitive clinical recommendations can be made.

### Limitations

#### Power and sample size

Although the sample size was prospectively calculated for the primary longitudinal within-patient outcome, it was based on expected physiological changes in Xc, as no previous studies comparing prosthetic designs using L-BIA were available. Consequently, the study was not specifically powered to detect between-group differences. Dividing the cohort into two randomized groups of 15 patients may therefore have limited the statistical power to detect subtle differences between prosthetic designs, increasing the risk of Type II error. In addition, 10 of the 40 randomized participants (25%) were not included in the final analysis because they did not receive the allocated intervention, were lost to follow-up, or had missing primary outcome data. Accordingly, the primary analyses were performed using a complete-case approach. This may have introduced attrition bias if participants not included in the final analysis differed systematically from those who completed follow-up.

Importantly, the study was designed as an exploratory superiority trial and was not powered or designed to establish equivalence or non-inferiority. Therefore, the absence of statistically significant between-group differences should not be interpreted as evidence of clinical equivalence between MP and UC implants, but rather as an absence of detectable differences within the precision and sample size of the present study. Larger, adequately powered comparative studies are required to determine whether clinically meaningful differences between implant designs exist.

Furthermore, no adjustment for multiple comparisons was applied to between-group and other exploratory analyses. Combined with the relatively small sample size and the number of secondary analyses, this increases the risk of false-positive findings. Therefore, individual statistically significant findings from these secondary analyses should be interpreted as exploratory.

#### Other limitations

Although no statistically significant between-group differences were detected in baseline demographic, clinical, or whole-body bioimpedance characteristics, some baseline imbalances were present. In addition, potentially relevant factors such as preoperative quadriceps strength, physical activity level, and rehabilitation potential were not formally assessed. In addition, L-BIA was not validated against direct measures of muscle strength or morphology, and objective functional performance tests were not included. Also, analyses were performed on a complete-case basis rather than according to the intention-to-treat principle, which may have introduced attrition bias and reduced the benefits of the original randomization. Finally, the comparison involved two specific implant systems, and the single-centre design with 12 months of follow-up may limit the generalizability of the findings.

## Conclusion

MP and UC implants showed no significant differences in quadriceps bioelectrical muscle recovery, clinical outcomes, or radiographic parameters during the first postoperative year.

L-BIA provided objective information on pre- and postoperative muscle tissue characteristics and did not identify significant differences in postoperative bioelectrical changes between implant groups. Baseline reactance was associated with postoperative reactance values, whereas no significant association was observed between implant design and postoperative L-BIA outcomes.

Given the exploratory nature and limited sample size of this randomized study, these findings should not be interpreted as evidence of equivalence between implant designs, and larger, adequately powered studies are needed to determine whether clinically meaningful differences in postoperative muscle recovery exist between MP and UC implants.

## Supplementary Information

Supplementary Material 1.

## Data Availability

The datasets analyzed during the current study are available from the corresponding authors on reasonable request.

## Abbreviations

TKA: Total Knee Arthroplasty
UC: Ultracongruent
MP: Medial Pivot
WOMAC: Western Ontario and McMaster Universities Osteoarthritis Index
KSS: Knee Society Score
L-BIA: Localized Bioimpedance Analysis
CR: Cruciate-retaining
PS: Posterior-stabilized
PCL: Posterior Cruciate Ligament
R: Resistance
Xc: Reactance
PhA: Phase angle
BMI: Body mass index
ASA: American Society of Anesthesiologists Classification
ROM: Range of motion
JL: Joint line
PH: Patellar Height
PCOR: Posterior condylar offset ratio
TS: Tibial slope
RF: Rectus femoris
VM: Vastus medialis
VL: Vastus lateralis
SD: Standard deviation
CI: Confidence interval
IQR: Interquartile range
