# Supplementary material for: Quadriceps muscle status after medial-pivot versus ultracongruent total knee arthroplasty using localized bioimpedance analysis: a randomized exploratory study
Source: Arthroplasty. 2026 Sep 7;8:68. doi: 10.1186/s42836-026-00429-z (PMC13548700; doi:10.1186/s42836-026-00429-z)
Supplement: Supplementary file 1 [file 42836_2026_429_MOESM1_ESM.docx]

**Supplementary material**

**Table S1.** Sensitivity analyses using ANCOVA adjusted for baseline Xc L-BIA, sex and BMI

| **Muscle** | **Predictor** | **Adjusted estimate (β)** | **95% CI** | ***p*-value** |
| --- | --- | --- | --- | --- |
| **Rectus femoris** | Implant type (UC vs MP) | −0.41 | −1.65 to 0.83 | 0.50 |
|  | Sex (Men vs Women) | 1.17 | −0.41 to 2.75 | 0.14 |
|  | BMI | −0.03 | −0.16 to 0.10 | 0.68 |
|  | Baseline Xc | 0.61 | 0.28–0.93 | <0.01* |
| **Vastus medialis** | Implant type (UC vs MP) | −0.70 | −2.28 to 0.88 | 0.37 |
|  | Sex (Men vs Women) | 0.77 | −1.37 to 2.91 | 0.46 |
|  | BMI | −0.05 | −0.21 to 0.12 | 0.55 |
|  | Baseline Xc | 0.94 | 0.58–1.31 | <0.01* |
| **Vastus lateralis** | Implant type (UC vs MP) | −1.31 | −3.06 to 0.44 | 0.14 |
|  | Sex (Men vs Women) | 0.44 | −1.78 to 2.66 | 0.69 |
|  | BMI | −0.10 | −0.28 to 0.09 | 0.30 |
|  | Baseline Xc | 0.95 | 0.63–1.27 | <0.01* |

Sensitivity analyses were performed using ANCOVA with implant type as the fixed factor and baseline Xc L-BIA, sex, and BMI included as covariates

* indicates statistical significance (*p* < 0.05)
